# Mitral Transcatheter Edge-to-Edge Repair for Anterior Leaflet Flail Induced by Transapical Transcatheter Aortic Valve Replacement

**DOI:** 10.1016/j.jaccas.2025.104653

**Published:** 2025-08-06

**Authors:** Matteo Betti, Sebastian A.F. Streukens, Suzanne Kats, Bart Maesen, Jindra Vainer, Ralph A.L.J. Theunissen, Arnoud W.J. van ‘t Hof, Pieter A. Vriesendorp

**Affiliations:** a Department of Cardiology, Cardiovascular Research Institute Maastricht, Maastricht University Medical Centre+, Maastricht, the Netherlands; b Department of Clinical Sciences and Community Health, Cardiovascular Section, University of Milan, Milan, Italy; c Department of cardiothoracic surgery, Cardiovascular Research Institute Maastricht, Maastricht University Medical Centre+, Maastricht, the Netherlands

**Keywords:** aortic, intervention, mitral, multivalve disease, transcatheter

## Abstract

**Background:**

Transapical transcatheter aortic valve replacement (TA-TAVR) is a viable option for inoperable patients who are not eligible for a transfemoral route. Rarely, delayed complications such as primary mitral regurgitation (MR) may emerge owing to procedural factors.

**Case Summary:**

An 85-year-old patient deemed at high surgical risk underwent TA-TAVR successfully for severe symptomatic aortic stenosis. Two months after the procedure, he returned with progressive dyspnea secondary to severe MR caused by anterior mitral leaflet flail. Mitral transcatheter edge-to-edge repair (M-TEER) was performed with a significant reduction in MR severity and notable symptomatic improvement.

**Discussion:**

This case describes an unusual late-onset complication of TA-TAVR. Early recognition and reintervention were key, as M-TEER offered a safe and effective strategy for treating this atypical complication.

**Take-Home Message:**

Severe primary MR can be a complication of TA-TAVR that may not be detectable in the immediate postoperative period, and M-TEER can be a valid option for its treatment.

## Visual Summary

**Figure:**
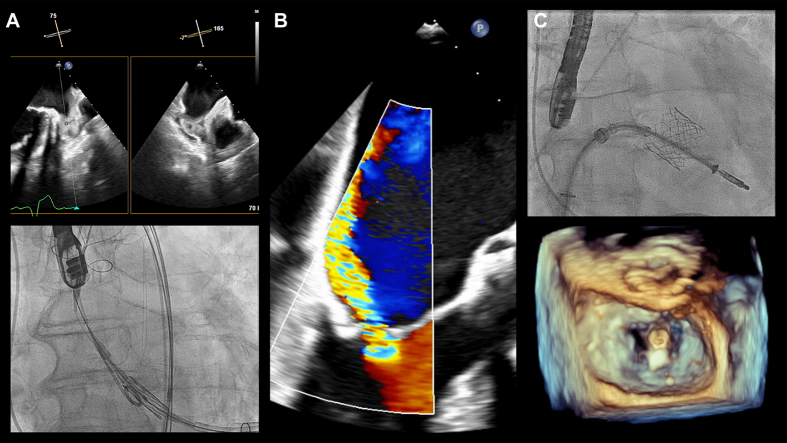

**Visual Summary:**
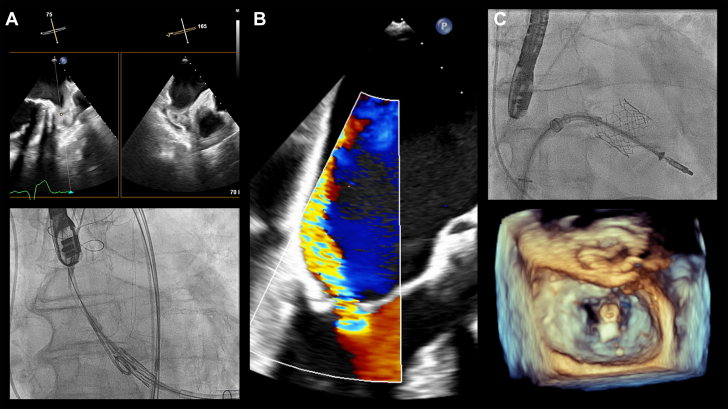
Transcatheter Interventions for Aortic and Mitral Valve Disease in an 85-Year-Old Patient (A) Transapical transcatheter aortic valve replacement was performed with the use of a cerebral embolic protection device owing to the presence of thrombus in the left atrial appendage. Technical and device success were achieved. (B) After 2 months, severe primary mitral regurgitation secondary to A2 flail was detected with the onset of new symptoms. (C) Mitral transcatheter edge-to-edge repair was performed with a single central (A2-P2) clip implantation, achieving mitral regurgitation reduction to grade 1.

## History of Presentation

An 85-year-old man presented to our hospital with severe dyspnea and chest pain on light exertion. He was already in follow-up for moderate aortic stenosis. Transthoracic echocardiography showed progression of the aortic disease with a low-flow low-gradient aortic stenosis, moderately reduced left ventricular ejection fraction, and mitral annular calcification (MAC) with mild mitral regurgitation (MR).

**Take-Home Messages**

• Severe primary MR can be a complication of TA-TAVR that may not be detectable immediately post-procedure.
• Dedicated transthoracic echocardiography should be performed with a search pattern for mitral valve disease in patients with recurrent symptoms post TA-TAVR.
• M-TEER can be a valid option for the treatment of degenerative mitral valve disease as a rare complication of TA-TAVR.

## Past Medical History

The patient had multiple risk factors (hypertension, diabetes mellitus type 2) and comorbidities (previous cerebrovascular accident, chronic atrophic gastritis, chronic kidney disease, peripheral arterial disease). He was known to our center for coronary artery disease with multiple previous percutaneous coronary interventions and for transcatheter ablation of atrial fibrillation. The patient was already on optimal medical therapy.

## Differential Diagnosis

The differential diagnosis included severe symptomatic aortic stenosis and chronic coronary syndrome.

## Investigations

**Figure 1:**
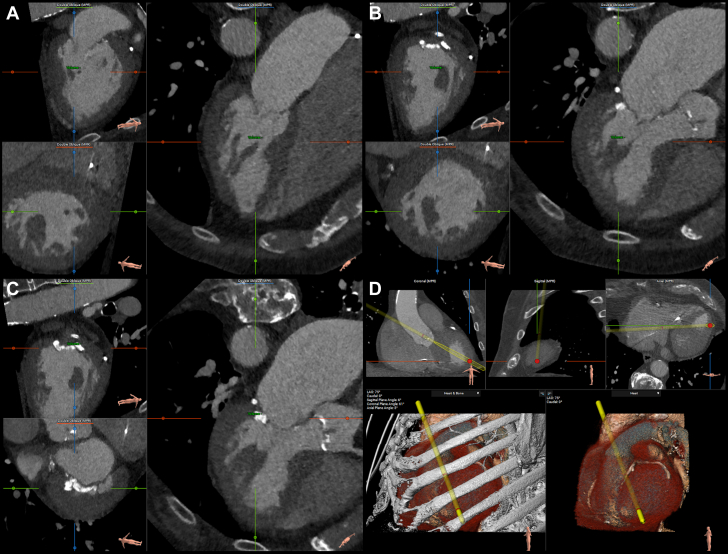
Preprocedural CTA Illustration of the anatomic characteristics of the left ventricle on preprocedural computed tomography angiography (CTA). (A and B) Multiplanar reconstructions of the left ventricle showing bifid anatomy of both the posteromedial (A) and the anterolateral (B) papillary muscle. (C) Multiplanar reconstruction of the left ventricle showing the mitral annular calcification. (D) Planning of the left ventricular puncture site.

Coronary angiography demonstrated unobstructed coronary arteries. The patient was discussed by the multidisciplinary heart team; he was declined for surgical aortic valve replacement owing to unacceptable operative risk and was judged unsuitable for transfemoral transcatheter aortic valve replacement (TAVR) because of poor arterial access, as minimal iliac-femoral artery diameter was 3 to 4 mm on both sides on computed tomography angiography (CTA). A final consensus toward combined transapical transcatheter aortic valve replacement (TA-TAVR) and thoracoscopic left atrial appendage (LAA) closure was reached. Transapical access is the preferred secondary access at our site, with extensive experience and good outcomes. Preprocedural CTA confirmed concentric hypertrophy of the left ventricle, with a normal internal diameter, and the MAC; interestingly, it also showed double bifid morphology of the papillary muscles (Figures 1A to 1D).

## Management (Medical/Interventions)

The initial procedure was aborted because a large thrombus was detected in the LAA at periprocedural transesophageal echocardiography (TEE). Of note, MR severity was judged as trivial (grade 0). The risk of cerebrovascular accidents was considered too high to proceed with either TAVR or LAA closure, so the patient was discharged on oral anticoagulant therapy. TEE performed at 3 months showed persistence of thrombus in the LAA. Therefore, a new multidisciplinary consultation recommended TA-TAVR with the use of a cerebral embolic protection device (EPD). The added benefit of the transapical route was the possibility of using femoral access for cerebral protection with the Flower device (AorticLab), as the patient was deemed not suitable for other commercially available devices using a radial approach (such as Sentinel EPD [Boston Scientific]).

Following left anterolateral mini-thoracotomy and preparation of the left ventricular apex, the apex was punctured with an 18G Seldinger needle, and the aortic valve was wired antegrade with a 0.035-inch wire under fluoroscopy without any issue or resistance. The needle was removed, and a 6-F JR4 diagnostic catheter was used to deliver an Amplatz Super Stiff wire in the descending aorta. After removal of the JR4, a 26-mm Edwards Sapien 3 Ultra device was introduced through a 21-F transapical delivery sheath and encountered some resistance crossing the valve (Video 1). Nevertheless, the delivery system was confirmed to be free from entanglement by TEE, so the valve was deployed by slow balloon inflation under rapid pacing (Video 2). The procedure ended without complications. Final TEE scans documented persistence of thrombus in the LAA and a normal mitral valve anatomy with trivial MR. An inspection of the EPD showed calcium debris but no thrombus.

**Figure 2:**
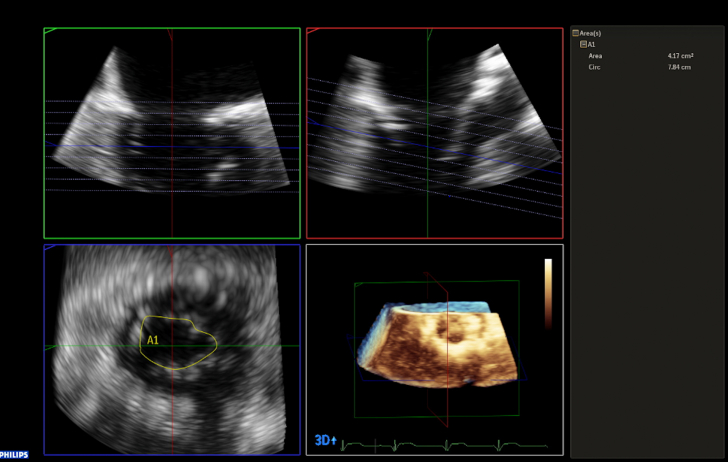
Assessment of Mitral Transcatheter Edge-to-Edge Repair Feasibility (Part 1) Measurement of mitral valve area by three-dimensional multiplanar reconstruction on transesophageal echocardiography.

**Figure 3:**
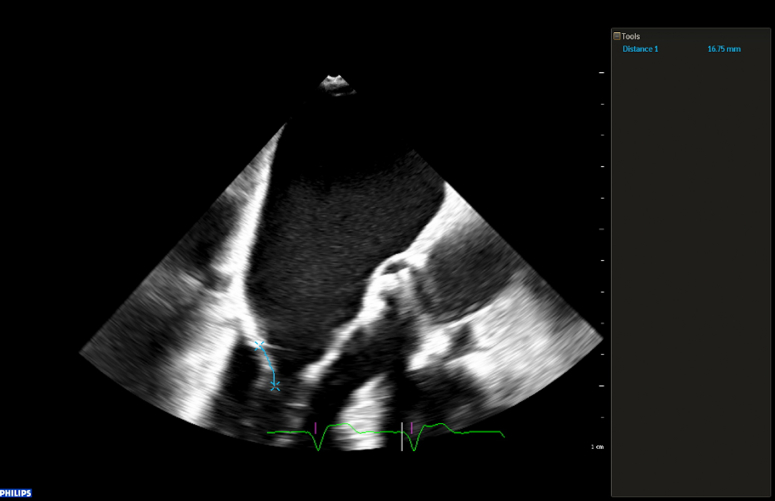
Assessment of M-TEER Feasibility (Part 2) Three-chamber view on transesophageal echocardiography illustrating the relationship between mitral annular calcification and the posterior leaflet; in particular, calcium extends to the base of P2, but the portion free of calcium had sufficient length (16 mm) to allow potential leaflet grasping during mitral transcatheter edge-to-edge repair (M-TEER).

**Figure 4:**
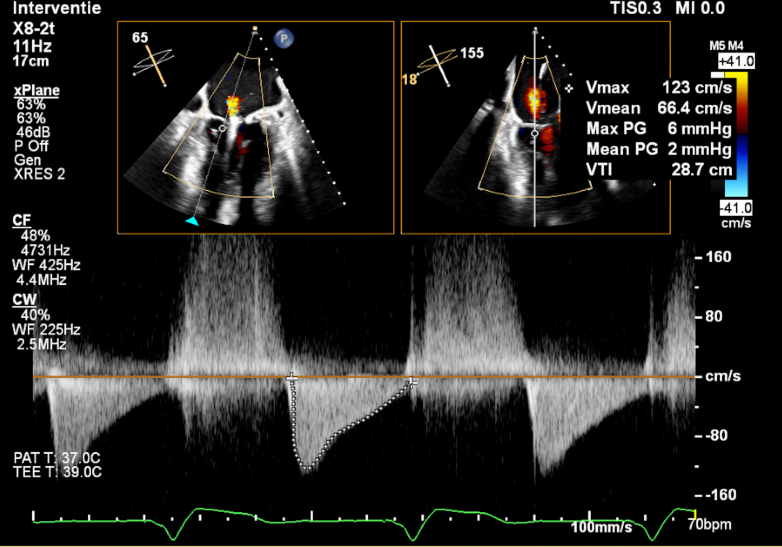
Assessment of Transmitral Postprocedural Gradient Transesophageal echocardiography showing normal transmitral gradient (2 mm Hg) after mitral transcatheter edge-to-edge repair.

At the 2-month follow-up, the patient reported an improvement of symptoms in the first month followed by a progressive deterioration. Coronary angiography was repeated to rule out significant coronary artery disease, and transthoracic echocardiography revealed moderate-to-severe MR. Therefore, TEE was performed and showed severe MR (grade 4, effective regurgitant orifice area >0.4 mm^2^) owing to A2 flail, which was not present before (Videos 3 and 4). Despite the MAC, the mitral valve area was calculated as 4.1 cm^2^, and the mean transmitral gradient was 2 mm Hg (Figure 2); the calcium involved also the base of P2, but the length of the leaflet free from it was 16 mm (Figure 3). Thrombus in the LAA was now absent. After establishing the technical feasibility and considering the patient's symptoms, a decision was made to perform mitral transcatheter edge-to-edge repair (M-TEER). A transseptal height of 6 cm allowed good maneuverability of the MitraClip G4 steerable guide catheter; a single XT clip was implanted in A2-P2 with reduction of MR from grade 4 to 1, while the mean gradient remained 2 mm Hg (Figure 4, Video 5, Video 6, Video 7). The patient tolerated the procedure well.

## Outcome and Follow-Up

The patient was discharged the following day in good clinical condition and asymptomatic. At the 2-week follow-up, he described a considerable improvement of his symptoms.

## Discussion

**Figure 5:**
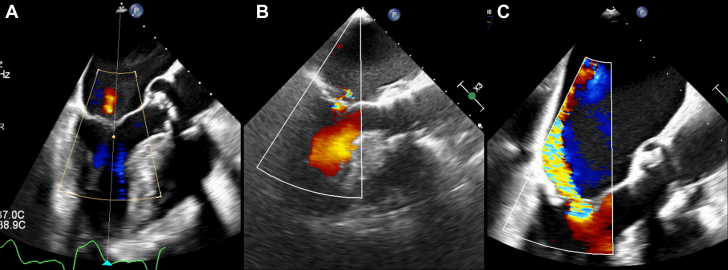
Timeline of MR Evolution Three-chamber view on transesophageal echocardiography illustrating the evolution of mitral regurgitation (MR) at 3 key time points. (A) Baseline, pre-transcatheter aortic valve replacement (TAVR): none/trivial MR. (B) Immediately post-TAVR: trivial MR. (C) 2-month follow-up after TAVR: severe primary MR.

We describe here a patient who presented with progressive heart failure symptoms 2 months after TA-TAVR owing to severe primary MR, which was successfully treated with M-TEER. TA-TAVR is a valid alternative for high-risk patients with extensive peripheral arterial disease and no surgical options. Although the transapical route may carry technical advantages over the transfemoral route, it entails unique procedural challenges that may lead to unexpected complications.^1^ Notably, acute severe MR can be induced by entanglement of the guidewire or delivery system with the mitral chordae, and it is usually associated with hemodynamic deterioration necessitating their retraction and repositioning.^2^ Although resistance to advancing the delivery system was felt (Video 1), we did not observe either clinical or echocardiographic signs of entanglement during the procedure. Other authors have reported iatrogenic MR as a periprocedural complication of transfemoral TAVR that required surgical treatment before discharge: they described a migration of a self-expanding prosthesis into the left ventricle during the self-expansion process, which required the implantation of a second self-expanding valve and led during the following days to severe MR owing to multiple ruptures of the mitral valve chordae and hemolysis, necessitating open heart surgery.^3^ In our case, the transcatheter valve remained stable when inflated under rapid pacing to achieve a conventional 80:20 ratio, and at the end of the procedure MR was trivial. Notwithstanding, after an uneventful postoperative course and a sustained clinical benefit, our patient experienced progressive heart failure symptoms ensuing from severe MR owing to A2 flail. Our explanation for such a phenomenon is that a small mitral chord was presumably damaged during the transit of the delivery system in the left ventricle, and even though neither the rupture nor the MR was detectable during intraoperative TEE immediately after TAVR, the latter possibly because of the acute reduction of the left ventricular afterload, the damage eventually progressed over time owing to increasing hemodynamic stress on the subvalvular apparatus. Figure 5 illustrates the evolution of MR during 3 key time points—at baseline, immediately post-TAVR, and at 2-month follow-up when severe MR was first documented.

Given the high operative risk inherent in our patient, who had already been turned down for surgery before TAVR, percutaneous M-TEER was selected as a less invasive alternative to conventional surgery. This strategy aligns with the recommendations set forth in the 2021 ESC/EACTS Guidelines for the management of valvular heart disease, which advocate for transcatheter mitral repair in symptomatic patients with severe MR who are considered unsuitable for surgical repair or replacement.^4^

The management of these procedural complications requires meticulous preprocedural planning, including comprehensive imaging assessments to evaluate the feasibility of transcatheter therapies and to anticipate potential challenges. In our case, a thorough TEE examination confirmed anatomic suitability for M-TEER: despite a central pathology, a mitral valve area >4.0 cm^2^, and a small flail gap and width, a challenging factor was the MAC with partial involvement of the posterior mitral leaflet.^5^ Intraoperatively, careful assessments of posterior mitral leaflet grasping and clip stability before release led to an optimal MR reduction from grade 4 to 1 with 1 central MitraClip XT.

Preventive strategies to avoid mitral complications during TA-TAVR start with looking for anatomic risk factors on preoperative CTA, such as small left ventricular cavities and density of the mitral subvalvular apparatus, potentially including bifid anatomy of the papillary muscles. Procedural strategies comprise advancing equipment in the left ventricle under fluoroscopy and, if resistance is felt, checking for clinical or echocardiographic signs of entanglement. Of note, during this step, resistance is often encountered as a consequence of the stenotic aortic valve itself or the angled trajectory, and thus intraprocedural imaging is key. Lastly, serial echocardiographic follow-up post-TAVR is crucial for the evaluation of MR evolution, which is usually favorable but might also be unexpectedly adverse, thereby allowing timely intervention.^6^

**EQUIPMENT LIST:** Illustration of the Materials Used for the 2 Transcatheter Interventions

TAVR	M-TEER
Imaging • TEE (Philips Healthcare, USA) ○ X8 TEE probe	Imaging • TEE (Philips Healthcare, USA) ○ 8 TEE probe
Delivery system and other materials • 18G Seldinger needle • 0.035 J wire • Certitude delivery system with 21-French sheath (Edwards Lifesciences, USA) • Amplatz Super Stiff wire (Boston Scientific, USA) • Pressure line to transduce intracardiac pressure • 6-F JR4 100 cm diagnostic catheter (Medtronic, USA) • 5-F straight Pigtail 110 cm catheter (Medtronic, USA)	Access • Ultrasound machine (Philips Healthcare, USA) • Micropuncture needle and wire • 0.035 J wire and 8-F sheath • 2 Proglides (Abbott Vascular, USA) • Hi-Torque Supra Core stiff guide wire (Abbott Vascular, USA) • 18-F Cook sheath (Cook Medical, USA
Embolic protection device • FLOWer 28 mm embolic protection device (AorticLab, Italy) • 12-F sheath	Transseptal puncture • BRK transseptal needle (Abbott Vascular, USA) • SL-1 sheath (Abbott Vascular, USA) • 0.032 wire • Pressure line to transduce intracardiac pressure • Safari XS stiff wire (Boston Scientific, USA)
TAVR device • 26 mm Sapien 3 Ultra valve (Edwards Lifesciences, USA)	Mitral TEER device • MitraClip G4 system (steerable guide catheter [SGC] and clip delivery system [CDS]), XT clip (Abbott Structural Heart, USA)

## Conclusions

This report illustrates that although TA-TAVR is a valuable option for inoperable patients, it carries rare risks such as injury to the mitral subvalvular apparatus that may necessitate additional interventions. Ultimately, it underscores the need for heightened awareness of potential delayed mitral complications and for detection of multivalve disease in patients undergoing transcatheter therapies. By providing successful outcomes of M-TEER for treating severe primary MR induced by TA-TAVR, we demonstrated that transcatheter options are effective and complementary in left-sided multivalve disease when complications occur.

## Funding Support and Author Disclosures

The authors have reported that they have no relationships relevant to the contents of this paper to disclose.

## Appendix

Video 1 Antegrade Crossing of the Aortic Valve by the TAVR Delivery System This video illustrates the antegrade crossing of the native aortic valve by the transcatheter aortic valve replacement (TAVR) delivery system and the resistance that was felt during this maneuver.

Video 2 Implantation of the Edwards Sapien 3 Ultra Valve This video illustrates the deployment of the Edwards Sapien 3 Ultra valve by slow inflation under rapid pacing, with good stability of the system.

Video 3 Severe Primary MR on TEE This video shows severe mitral regurgitation (MR) secondary to anterior mitral leaflet flail in the 3-chamber view on transesophageal echocardiography (TEE), with the regurgitant jet directed toward the posterior wall of the left atrium.

Video 4 Three-Dimensional En-Face TEE View of the Mitral Valve Showing A2 Flail This video illustrates the mechanism of severe primary mitral regurgitation consisting of A2 flail on the three-dimensional surgical en-face transesophageal echocardiography (TEE) view of the mitral valve.

Video 5 Assessment of Clip Orientation on Intraprocedural TEE During Mitral Transcatheter Edge-to-Edge Repair This video shows the three-dimensional en-face view of the mitral valve on intraprocedural transesophageal echocardiography (TEE) to assess clip alignment and perpendicularity to the coaptation line before grasping.

Video 6 Closing of the Clip on Intraprocedural TEE During Mitral Transcatheter Edge-to-Edge Repair This video shows the intraprocedural two-dimensional transesophageal echocardiography (TEE) biplane (XPlane) bicommissural view (left) and left ventricular outflow tract view (right) to assess leaflet grasping and insertion before clip closure.

Video 7 Final Result After Mitral Transcatheter Edge-to-Edge Repair This video shows the intraprocedural two-dimensional transesophageal echocardiography biplane (XPlane) bicommissural view (left) and left ventricular outflow tract view (right) to assess residual MR after clip implantation. Residual MR is judged as mild (grade 1).

Equipment List ▪▪▪

## References

[1] Ye J., Cheung A., Lichtenstein S.V. (2010). Transapical transcatheter aortic valve implantation: follow-up to 3 years. J Thorac Cardiovasc Surg.

[2] Al-Attar N. Complications of transapical aortic valve implantation - an article from the e-journal of the ESC council for cardiology practice. https://www.escardio.org/Journals/E-Journal-of-Cardiology-Practice/Volume-9/Complications-of-transapical-aortic-valve-implantation.

[3] Ma X., Gong K., Zhao L., Chen X., Fu X. (2023). Mitral valve chordal rupture caused by prosthetic valve migration after transcatheter aortic valve implantation. CASE (Phila).

[4] Vahanian A., Beyersdorf F., Praz F., ESC/EACTS Scientific Document Group (2022). 2021 ESC/EACTS guidelines for the management of valvular heart disease. Eur Heart J.

[5] Hausleiter J., Stocker T.J., Adamo M., Karam N., Swaans M.J., Praz F. (2023). Mitral valve transcatheter edge-to-edge repair. EuroIntervention.

[6] Witberg G., Codner P., Landes U. (2021). Effect of transcatheter aortic valve replacement on concomitant mitral regurgitation and its impact on mortality. JACC Cardiovasc Interv.

