# Supplementary material for: Mitral Transcatheter Edge-to-Edge Repair for Anterior Leaflet Flail Induced by Transapical Transcatheter Aortic Valve Replacement
Source: JACC Case Rep. 2025 Aug 6;30(22):104653. doi: 10.1016/j.jaccas.2025.104653 (PMC12426552; doi:10.1016/j.jaccas.2025.104653)
Supplement: Equipment List — ▪▪▪ [file mmc8.docx]

| **Transcatheter Aortic Valve Replacement (TAVR)** | **Mitral Transcatheter Edge-to-Edge Repair (M-TEER)** |
| --- | --- |
| Imaging   - Tranesophageal echocardiography (TEE) (Philips Healthcare, USA)   - X8 TEE probe | Imaging   - Tranesophageal echocardiography (TEE) (Philips Healthcare, USA)   - X8 TEE probe |
| Delivery system and other materials   - 18G Seldinger needle - .035 J wire - Certitude delivery system with 21-French sheath (Edwards Lifesciences, USA) - Amplatz Super Stiff wire (Boston Scientific, USA) - Pressure line to transduce intracardiac pressure - 6-French JR4 100 cm diagnostic catheter (Medtronic, USA) - 5-French straight Pigtail 110 cm catheter (Medtronic, USA) | Access   - Ultrasound machine (Philips Healthcare, USA) - Micropuncture needle and wire - .035 J wire and 8-French sheath - 2 Proglides (Abbott Vascular, USA) - Hi-Torque Supra Core stiff guide wire (Abbott Vascular, USA) - 18-French Cook sheath (Cook Medical, USA |
| Embolic protection device   - FLOWer 28 mm embolic protection device (AorticLab, Italy) - 12-French sheath | Transseptal puncture   - BRK transseptal needle (Abbott Vascular, USA) - SL-1 sheath (Abbott Vascular, USA) - .032 wire - Pressure line to transduce intracardiac pressure - Safari XS stiff wire (Boston Scientific, USA) |
| TAVR device   - 26 mm Sapien 3 Ultra valve (Edwards Lifesciences, USA) | Mitral TEER Device   - MitraClip G4 system (steerable guide catheter (SGC) and clip delivery system (CDS)), XT clip (Abbott Structural Heart, USA) |

**Supplemental Figure 1. Equipment list**. Illustration of the materials used for the two transcatheter interventions.
